# Perturbation-Based Balance Exercise Using a Wearable Device to Improve Reactive Postural Control

**DOI:** 10.1109/JTEHM.2023.3310503

**Published:** 2023-08-31

**Authors:** Masataka Yamamoto, Koji Shimatani, Daiki Yoshikawa, Taku Washida, Hiroshi Takemura

**Affiliations:** Faculty of Science and TechnologyTokyo University of Science 26413 Noda Chiba 278-8510 Japan; Graduate School of Advanced Science and EngineeringHiroshima University 12803 Higashihiroshima 739-8527 Japan; Department of RehabilitationFukuyama Memorial Hospital Fukuyama 721-0964 Japan; Faculty of Health and WelfarePrefectural University of Hiroshima 12798 Mihara Hiroshima 723-0053 Japan

**Keywords:** External perturbation, reactive postural control, balance, standing

## Abstract

Reactive postural control is an important component of the balance function for fall prevention. Perturbation-based balance exercises improve reactive postural control; however, these exercises require large, complex instruments and expert medical guidance. This study investigates the effects of unexpected perturbation-based balance exercises using a wearable balance exercise device (WBED) on reactive postural control. Eighteen healthy adult males participated in this study. Participants were assigned to the WBED and Sham groups. In the intervention session, participants in the WBED group randomly underwent unexpected perturbation in the mediolateral direction, while the Sham group performed the same exercises without perturbation. Before and after the intervention session, all participants underwent evaluation of reactive balance function using air cylinders. Peak displacement (D), time at peak displacement (T), peak velocity (V), and root mean square (RMS) of center of pressure (COP) data were measured. For mediolateral and anteroposterior COP (COPML and COP $_{\mathrm {AP}}$), the main effects of group and time factors (pre/post) were investigated through the analysis of variance for split-plot factorial design. In the WBED group, the D-COPML and V-COPML of the post-test significantly decreased compared to those of the pre-test (p = 0.017 and p = 0.003, respectively). Furthermore, the D-COPAP and RMSAP of the post-test significantly decreased compared to those of the pre-test (p = 0.036 and p = 0.015, respectively). This study proved that the perturbation-based balance exercise using WBED immediately improved reactive postural control. Therefore, wearable exercise devices, such as WBED, may contribute to the prevention of falls and fall-related injuries.

## I. Introduction

Falls are a major health concern in older individuals because it decreases activities of daily living. Falls and related injuries also cause the risk of long-term admissions into nursing homes [1]. It has been reported that approximately 40% of older individuals fall at least once a year [2]. Falls and related injuries increase with an increasingly aging population. Postural control is the primary function for active control of body alignment and stabilization center of mass against external disturbances [3]. Postural control is a complex skill consisting of nine components: functional stability limits, motor systems, static stability, verticality, anticipatory postural control, dynamic stability, sensory integration, cognitive influences, and reactive postural control [4]. A decrease in postural control correlates with the risk of falls in older adults and people with chronic central nervous system diseases [5], [6]. The relative risk of balance impairment for falls is approximately 1.2–2.4 times higher in community-living older adults [7]. Balance exercise is an effective treatment for the prevention of falls. Increasing anteroposterior stability, which is the primarily used ankle joint strategy, is also effective for postural control. However, a decrease in postural control caused by aging has affected the mediolateral stability as well as anteroposterior stability [8]. Lateral muscle activity, such as gluteus medius and external oblique, increased when lateral perturbation occurs during standing [9]. Further, these muscle activities and associated joint moments were important for mediolateral stability during gait and single-leg stance with lateral leg lifts [10], [11]. Therefore, improving mediolateral postural control is important for fall prevention and extension of a healthy lifespan in older adults.

To maintain an upright posture against external perturbation, anticipatory postural adjustments (APAs) and compensatory postural adjustments (CPAs) are used by the central nervous system. APA is used as a feed-forward postural control that minimizes potential postural change before expected external perturbations occur [12], [13]. Contrarily, CPAs are used as corrective postural control functions after the external perturbation to restore the position of the body [14], [15]. APAs are important in reducing balance instability caused by external perturbation and reducing adjustment of CPAs; however, APAs cannot be used for unexpected perturbation. Therefore, reactive postural control in the CPA phase is important in maintaining the posture after unexpected external perturbation [9].

Reactive postural control is one of the most important components of balance function, which refers to the ability to recover stability after unexpected external perturbation through corrective body movement [4]. Reactive postural control may be considered a more challenging component than general static and dynamic balance functions. Compared to younger adults, older adults are less able to stabilize their center of pressure (COP) after unexpected perturbations. Moreover, reactive postural control is decreased in the older adults who have experienced falls compared with those who have not experienced falls [16]. Decreased reactive postural control increases the COP displacement, COP velocity, delayed reaction time to unexpected perturbation, and electromyography (EMG) activity [17], [18], [19]. Unexpected perturbation-based balance exercises improve balance functions, such as reaction time, and the functional balance test scores. Kurz et al. [20] reported that unexpected perturbation exercise during gait improved balance function and COP data related to risk factors of falls. Perturbation-based exercise using a balance-exercise-assist robot is also effective in improving the balance function and gait speed in frail or prefrail older adults [21]. Therefore, reactive postural control is particularly important in fall prevention. However, unexpected perturbation-based balance exercises are difficult to perform because they require large and expensive therapeutic instruments or medical-expert skills. To easily improve reactive postural control at home, it is necessary to develop a device that is lightweight, small or wearable, and automatically applicable in small-unexpected perturbations.

Recently, many wearable devices have been developed for human health and rehabilitation. Although these wearable devices were small and lightweight, they were also used for feedback on rehabilitation exercises, gait recovery prediction, and prevention of medical complications [22], [23], [24]. These devices can assist human movements and help with activities of daily living. Pneumatic artificial muscles (PAM) are useful actuators for wearable assistive or exercise devices being lightweight, flexible, and inexpensive. Thus, actuators are easy to use at home. Wearable assist devices using PAM increase the ankle range of motion and decrease muscle activity during gait [25], [26]. A wearable balance exercise device (WBED) for reactive postural control also uses PAM, which is developed to allow users to perform balance exercises at home. This device is flexible, lightweight, and easy to use at home. PAMs are used for generating unexpected perturbations. A previous study reported that WBED can be used as a device for balance exercise, and it immediately improves the static balance function [27]. Although this device allows easy perturbation-based balance exercises at home, the effect of WBED on reactive postural control remains unclear. Reactive postural control should be simultaneously improved with the static balance function. If individuals can improve reactive postural control in their home using a device such as WBED, it might contribute to the prevention of falls and fall-related injuries. Therefore, this study aimed to investigate the effects of perturbation-based balance exercises performed using WBED on reactive postural control.

## II. Methods

### A. Participants

Twenty healthy young adult males (age: 23.0 ± 0.91) participated in this study. The exclusion criteria were as follows: age < 20 years, no surgery in the year before study participation, history of neurological disorders or cardiac disease, and no back or lower limb pain during standing or gait. All study procedures were approved by the ethics committee of the Tokyo University of Science (21021), and written informed consent was obtained from all participants before the experiment. Power analysis was used to determine the sample size (alpha level: 0.05, power: 0.80, and effect size: 0.40) in this study; the required total sample size was 28. The number of participants in this study was less than the required sample size; therefore, post-hoc analysis for calculating the achieved power was performed based on the results of this study.

### B. Wearable Balance Exercise Device (Wbed)

**Fig. 1.**
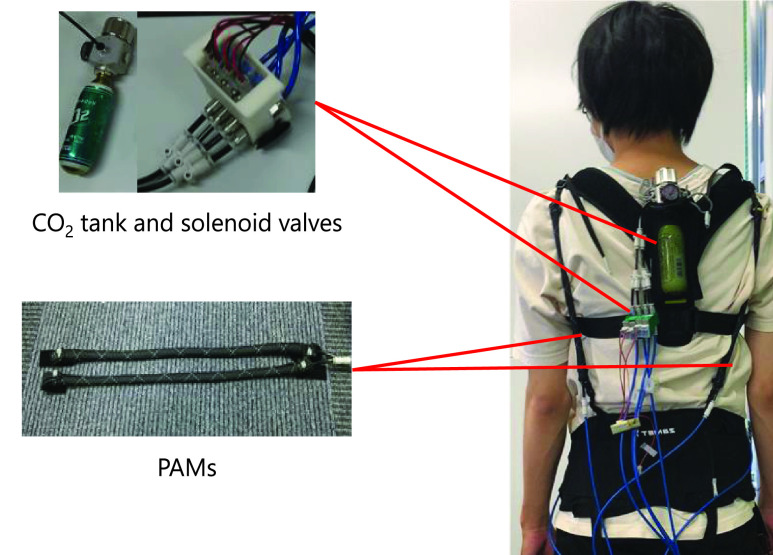
Configuration of the WBED. The PAMs contraction was controlled these solenoid valves according to the control command from smartphone. These PAMs generated contraction force in the direction of lateral trunk bending.

**Fig. 2.**
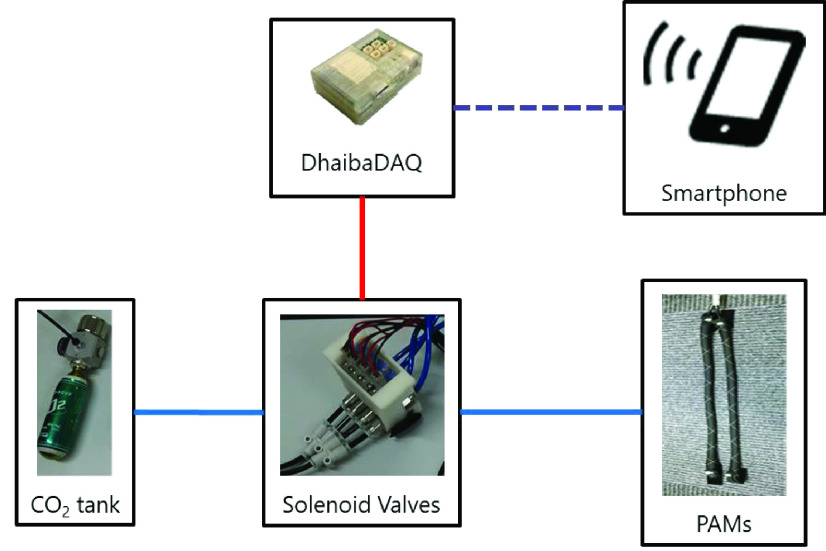
System configuration of PAM contraction using DhaibaDAQ. Wi-fi connecting is represented by black dashed line. Electronic circuit is represented by red line. Pneumatic flow for PAMs is represented by blue line.

WBED was used for perturbation-based reactive balance exercises in this study (Fig. 1). The WBED consisted of a soft supporter, soft pelvic belt, solenoid valves, CO_2_ tank, and four McKibben-type PAMs. It was designed to be lightweight, portable, and easy to use at home and in clinical settings. The WBED weighed 0.9 kg, and it took less than 3 min to wear the device. The soft supporter and pelvic belt were used to support and affix the four PAMs. This device can generate unexpected small perturbations and improve standing stability [13]. In this study, four PAMs with a natural length of 250 mm were extended by 290 mm and attached to a WBED. Two PAMs were attached to both the right and left sides of the WBED. These side PAMs generated a force in the direction of lateral trunk bending. The PAMs were connected to the CO_2_ tank (mini gas cylinder, NTG, Tokyo, Japan) using solenoid valves (SYJ300, SMC, Tokyo, Japan). A DhaibaDAQ module was used for automated control of PAMs. DhaibaDAQ is small, light, and can easily control PAMs by a smartphone [28]. When the solenoid valve was opened by the DhaibaDAQ and smartphone (ASUS_Z00ED, ASUS, Taipei, Taiwan), the PAM contracted due to compressed air flow from the CO_2_ tank. From the experimental test, a single PAM generated 20 N in a 0.2 MPa air pressure condition. The system configuration of PAM contraction is shown in Fig. 2.

### C. Experimental Setup and Procedure

**Fig. 3.**
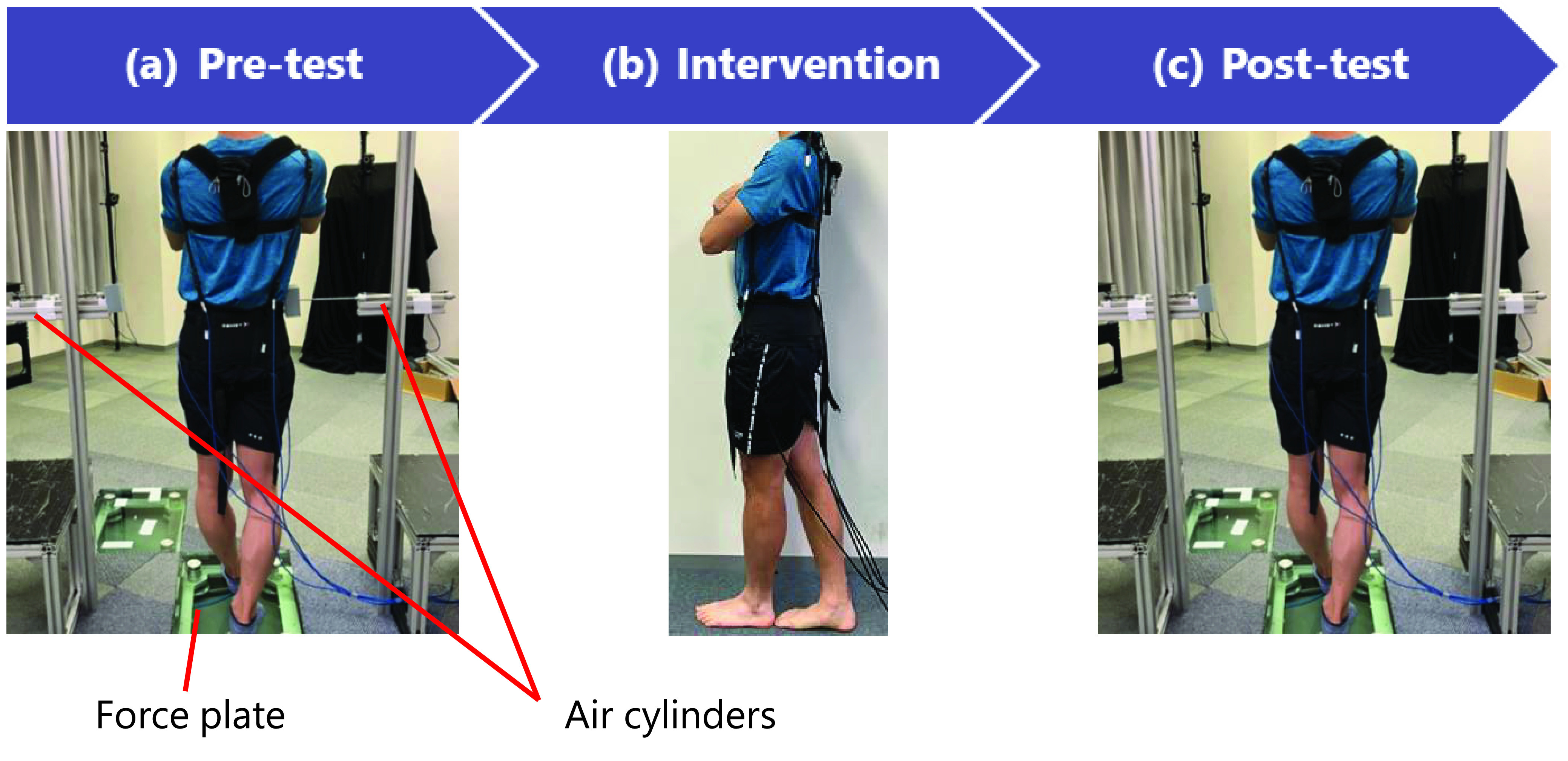
Overview of the experimental procedure in this study. The experimental procedures were performed in the order of pre-test (a), Intervention (b), and post-test (c). (a) and (c): participants were asked to perform tandem stance on the force plate. Air cylinders perturbed the subjects laterally during tandem stance. (b): The intervention consists of 16 tandem stance exercises in total. The participants in WBED group were perturbed by WBED during tandem stance, while the participants in Sham group were not perturbed.

An overview of the experimental procedure is shown in Fig. 3. The experiment was conducted in the following order: pre-test, intervention, and post-test. Participants were randomly assigned to the WBED and Sham groups. Before the intervention, all participants underwent a pre-test for verifying the effect of the intervention on reactive postural control. Two air cylinders (CJ2E16-200AZ, SMC, Tokyo, Japan) were used for unexpected lateral perturbations refer to previous studies [29]. In the pre-test, the participants were asked to perform a tandem stance with the dominant leg behind, on the force plate (Tech Gihan, Kyoto, Japan), and maintain their balance as consistently as possible for a minute. The dominant leg was defined as the leg that participants preferred to use to kick a ball [30]. Two air cylinders were placed on each side at the height of the iliac crests of the participant. These air cylinders laterally perturbed pelvis of the participants with approximately 88 N during the pre-test. As the lateral perturbation using the air cylinders was performed three times at random timings, the participants could not expect the perturbations in the pre-test.

After the pre-test, the participants were asked to perform four sessions of tandem stance exercises. Each session consisted of four one-minute tandem exercises. During the intervention session, participants looked at a sign 2 m away, which was placed at a height corresponding to the height of their eyes. In the WBED group, participants randomly underwent unexpected perturbation in the mediolateral direction by the PAMs. Although the Sham group participants performed the same exercises while wearing the WBED, the PAMs did not contract during the intervention session. Finally, the post-test was conducted in the same manner as the pre-test. Participants were allowed to rest at any time during the experiment. An experimental collaborator was near the participants for fall prevention. Handrails were also set up on both sides of the participant.

### D. Data Analysis and Statistical Analysis

Time series COP data of the pre-test and post-test were collected with a 100 Hz sampling frequency. Zero-phase, low-pass Butterworth filter with a 10 Hz cut-off frequency was used for the COP data. The timing of the electrical trigger to open the solenoid valve for the air cylinders and the time until the air cylinder contacted the pelvis of the participants were used to identify the timing of the perturbations (time = 0) in the pre-test and post-test. The baseline of mediolateral and anteroposterior COP (COP_ML_ and COP $_{\mathrm {AP}}$) was calculated using the mean value from −0.35 to −0.2 s. The mean values were subtracted from the COP_ML_ and COP_AP_ time series data. To assess the effect of interventions on reactive postural control, peak displacement (D-COP), time at D-COP (T-COP), peak velocity (V-COP), and root mean square (RMS) of COP_AP_ and COP_ML_ were calculated from 0–0.4 s, referred to previous studies [31], [32]. These COP data were used as representative parameters for assessing reactive postural control. Peak D-COP was quantified as the maximum change in the same direction as the force by the air cylinder between 0–0.4 s. Previous studies reported that trunk and leg muscle, including lateral side muscles, activity detected in response to external perturbation was found in that time window [33], [34]. The force plate and air cylinders were synchronized using an electrical trigger.

To compare these COP parameters, the main effects of group (WBED/Sham) and time factors (pre-test/post-test) were investigated by analyzing the variance for split-plot factorial design. Post-hoc tests with Shaffer’s modified sequentially rejective Bonferroni procedure were performed to determine between the group and time factors. These statistical analyses were performed using R (version 4.2.1; CRAN, freeware). The partial eta-squared $\eta ^{2}$: small $>$ 0.01, moderate $>$ 0.06, or large $>$ 0.14) was used as the effect size. Statistical significance was set at p < 0.05. Moreover, a post-hoc analysis for calculating the achieved power was performed based on the results of this study.

## III. Results

**Fig. 4.**
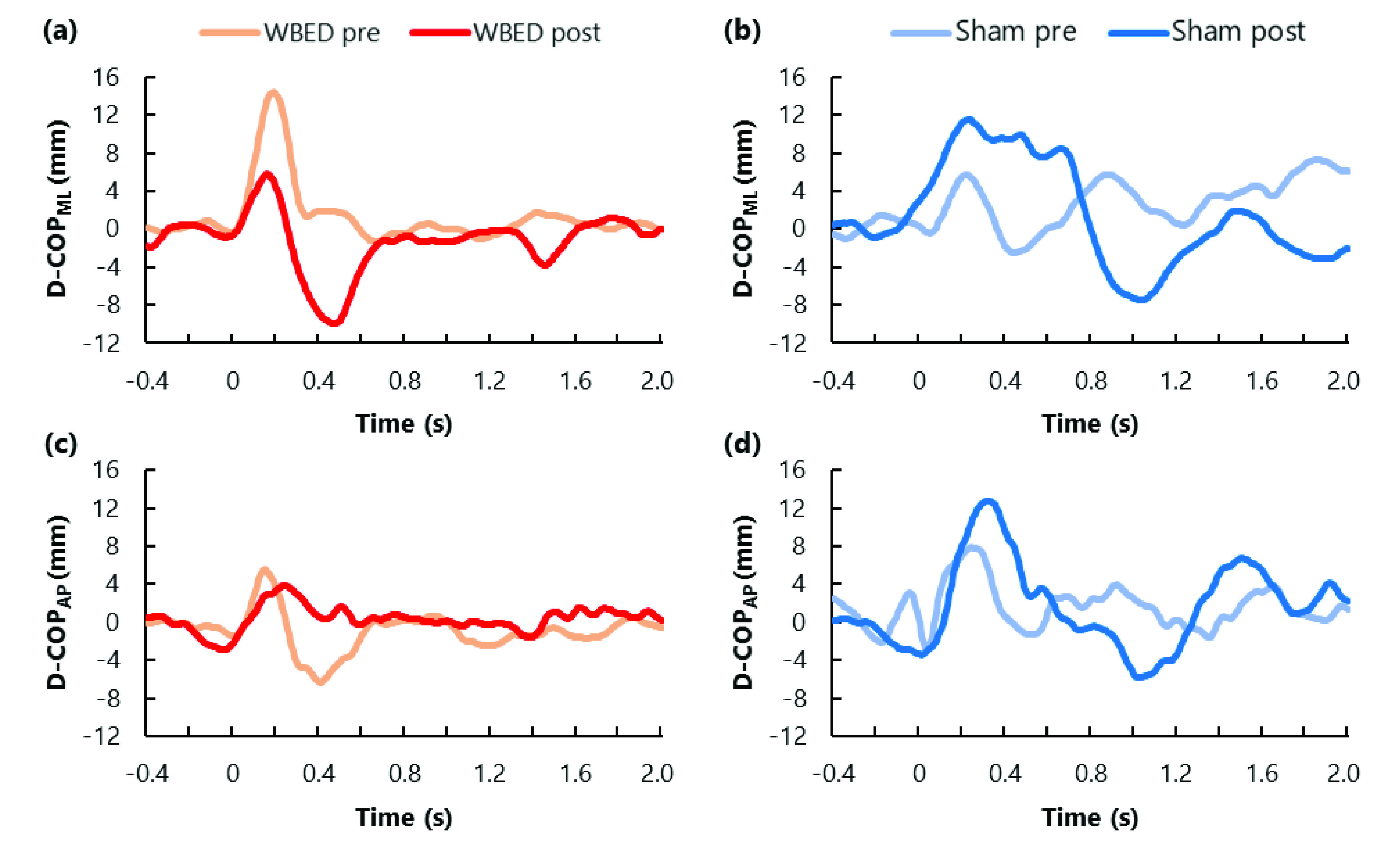
Example of the temporal change of D-COP on WBED group (left side) and Sham group (right side). Time 0 means the timing of the perturbation by the air cylinder. COP_ML_ displacement in WBED group (a), COP_ML_ displacement in Sham group (b), COP_AP_ displacement in WBED group (c), and COP_AP_ displacement in Sham group (d). D-COP_ML_ change in the same direction as force direction by the air cylinder and anterior of D-COP_AP_ are defined as positive.

**Fig. 5.**
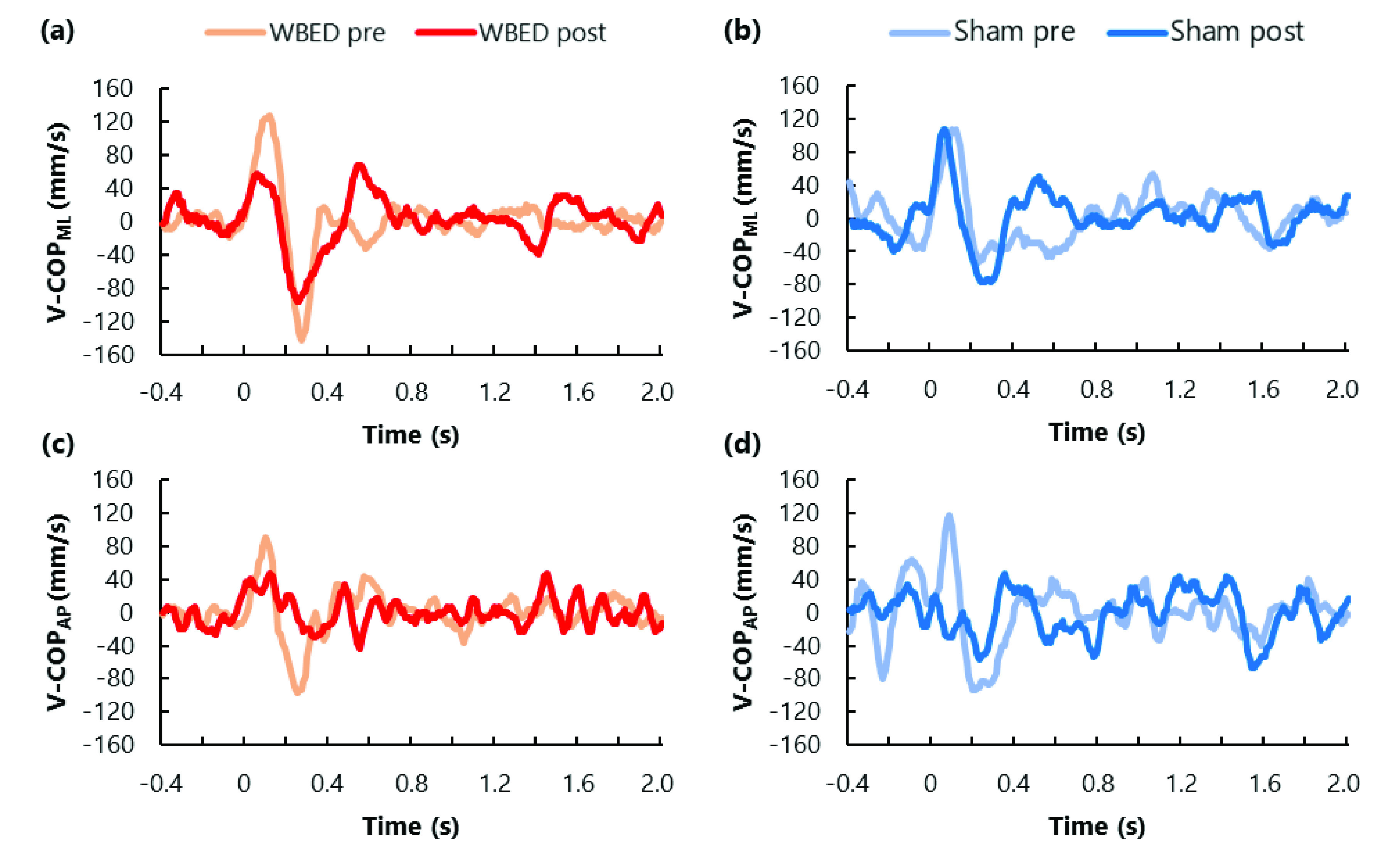
Example of the temporal change of V-COP on WBED group (left side) and Sham group (right side). Time 0 means the timing of the perturbation by the air cylinder. COP_ML_ velocity in WBED group (a), COP_ML_ velocity in Sham group (b), COP_AP_ velocity in WBED group (c), and COP_AP_ velocity in Sham group (d). V-COP_ML_ change in the same direction as force direction by the air cylinder and anterior of V-COP_AP_ are defined as positive.

Representative temporal changes in D-COP and V-COP from −0.4 to 2.0 s for each group and time factor are shown in Fig. 4 and 5. The mean ± standard deviation (SD) of each COP parameter is presented in Tables 1 and 2. Data from 18 participants were used in this study because the COP data of two participants could not be sufficiently measured. Post-hoc analysis for calculating achieved power was conducted based on the effect size of the main effect, which was significantly different in the analysis of variance for split-plot factorial design; the smallest power was 0.934.

**TABLE 1:** Results for COP_ML_ parameters

			Effect of group	Effect of time	Interaction
Group	D-COP $_{\mathbf {ML}}$(mm)		F = 0.049, p = $0.828\,\,\eta _{\mathrm {p}}^{2}=0.030$	F = 6.074, p = $0.025\,\,\ast \,\,\eta _{\mathrm {p}}^{2}=0.275$	F = 1.972, p = $0.179\,\,\eta _{\mathrm {p}}^{2}=0.110$
	Pre	Post			
WBED	15.4 ± 10.9	6.6 ± 5.5 y			
Sham	11.6 ± 8.2	9.2 ± 6.2			
	T-COP $_{\mathbf {ML}}$(ms)		F = 0.264, p = $0.614\,\,\eta _{\mathrm {p}}^{2}=0.016$	F = 0.440, p = $0.516\,\,\eta _{\mathrm {p}}^{2}=0.027$	F = 3.607, p = $0.076\,\,\eta _{\mathrm {p}}^{2}=0.184$
	Pre	Post			
WBED	217.8 ± 76.4	187.8 ± 101.5			
Sham	157.8 ± 33.5	220.0 ± 80.5			
	V-COP $_{\mathbf {ML}}$(mm/s)		F = 0.612, p = $0.445\,\,\eta _{\mathrm {p}}^{2}=0.037$	F = 11.735, p = $0.004\,\,\ast \ast \,\,\eta _{\mathrm {p}}^{2}=0.423$	F = 3.613, p = $0.076\,\,\eta _{\mathrm {p}}^{2}=0.184$
	Pre	Post			
WBED	166.7 ± 74.1	69.6 ± 33.0 yy			
Sham	115.6 ± 70.4	87.8 ± 49.3			
	RMS $_{\mathbf {ML}}$(mm)		F = 0.190, p = $0.669\,\,\eta _{\mathrm {p}}^{2}=0.012$	F = 2.232, p = $0.155\,\,\eta _{\mathrm {p}}^{2}=0.122$	F = 1.091, p = $0.312\,\,\eta _{\mathrm {p}}^{2}=0.064$
	Pre	Post			
WBED	11.0 ± 7.7	6.3 ± 3.0			
Sham	8.2 ± 7.0	7.3 ± 4.5			

These values were represented as mean ± SD. $\ast $ and $\ast \ast $ indicate significant difference at main effect (p < 0.05 and p < 0.01, respectively). yand yyindicate significant difference at the same group (p < 0.05 and p < 0.01, respectively).

**TABLE 2:** Results for COP_AP_ parameters

			Effect of group	Effect of time	Interaction
Group	D-COP $_{\mathbf {AP}}$(mm)		F = 3.976, p = $0.064\,\,\eta _{\mathrm {p}}^{2}=0.199$	F = 6.604, p = $0.021\,\,\ast \,\,\eta _{\mathrm {p}}^{2}=0.292$	F = 0.597, p = $0.451\,\,\eta _{\mathrm {p}}^{2}=0.036$
	Pre	Post			
WBED	14.0 ± 7.7	9.0 ± 6.8 y			
Sham	7.6 ± 4.9	4.9 ± 5.8			
	T-COP $_{\mathbf {AP}}$(ms)		F = 0.645, p = $0.434\,\,\eta _{\mathrm {p}}^{2}=0.039$	F = 0.001, p = $0.973 \eta _{\mathrm {p}}^{2}< 0.001$	F = 0.388, p = $0.542\,\,\eta _{\mathrm {p}}^{2}=0.024$
	Pre	Post			
WBED	206.7 ± 103.4	187.8 ± 42.7			
Sham	163.3 ± 84.7	184.4 ± 118.5			
	V-COP $_{\mathbf {AP}}$(mm/s)		F = 1.293, p = $0.272\,\,\eta _{\mathrm {p}}^{2}=0.075$	F = 3.564, p = $0.077\,\,\eta _{\mathrm {p}}^{2}=0.182$	F = 0.086, p = $0.773\,\,\eta _{\mathrm {p}}^{2}=0.005$
	Pre	Post			
WBED	244.4 ± 145.0	157.8 ± 183.7			
Sham	171.9 ± 116.8	108.5 ± 108.1			
	RMS $_{\mathbf {AP}}$(mm)		F = 0.964, p = $0.341\,\,\eta _{\mathrm {p}}^{2}=0.057$	F = 7.602, p = $0.014\,\,\ast \,\,\eta _{\mathrm {p}}^{2}=0.322$	F = 2.357, p = $0.144\,\,\eta _{\mathrm {p}}^{2}=0.128$
	Pre	Post			
WBED	8.4 ± 4.4	5.0 ± 3.6 y			
Sham	5.9 ± 2.6	4.9 ± 2.5			

These values were represented as mean ± SD. $\ast $ and $\ast \ast $ indicate significant difference at main effect (p < 0.05 and p < 0.01, respectively). yand yyindicate significant difference at the same group (p < 0.05 and p < 0.01, respectively).

### A. COP_ML_ PARAMETERS

There were significant main effects of time for D-COP_ML_ and V-COP_ML_, and both effect sizes were large. No statistically significant differences were found in the main effects of the group factors for these COP_ML_ parameters. Additionally, this interaction was not significant. In the WBED group, the D-COP_ML_ and V-COP_ML_ of the post-test significantly decreased compared to those of the pre-test (p = 0.017 and p = 0.003, respectively). However, no statistically significant differences were found in the D-COP_ML_ and V-COP_ML_ of the Sham group between the pre-test and post-test (p = 0.508 and p = 0.418, respectively).

### B. COP_AP_ PARAMETERS

There were significant main effects of time factor for D-COP_AP_ and RMS_AP_, and both effect sizes were large. No statistically significant differences were found in the main effects of the group factors for these COP_AP_ parameters. Furthermore, this interaction was not significant. In the WBED group, the D-COP_AP_ and RMS_AP_ of the post-test significantly decreased compared to those of the pre-test (p = 0.036 and p = 0.015, respectively). No statistically significant differences were found in the D-COP_AP_ and RMS_AP_ of the Sham group between the pre-test and post-test (p = 0.265 and p = 0.362, respectively).

## IV. Discussion

Improving reactive postural control ability is critical for fall prevention. Further, improving mediolateral stability contributes to the stability of standing and gait. This study aimed to clarify the effects of unexpected perturbation-based balance exercises using WBED on reactive postural control. The experimental results showed that balance exercises using WBED immediately improved reactive postural control.

Furthermore, the D-COP_ML_ owing to the unexpected perturbation appeared from 0 to 400 ms. Previous studies reported peak COP displacement after unexpected perturbation was observed during this phase. Our results were consistent with those of previous studies. Among the COP_ML_ parameters of the WBED group, D-COP_ML_ and V-COP_ML_ of the post-test were significantly lower than those of the pre-test. Reactive postural control decreases with aging, and the experience of falls and fall risk increases with a decrease in reactive postural control. Compared to younger adults, older adults with and without experience of falls could not stabilize their COP after unexpected perturbations [16]. Reactive postural control during this phase serves to reduce the displacement of COP caused by unexpected perturbation. Perturbation-based balance exercises using a stand-up-and-ride instrument improve the functional base of support and tandem gait speeds [35]. Furthermore, waist-pull perturbation-based balance exercises during walking improve the response to perturbation and gait stability in Parkinson’s disease [36]. Central nervous system diseases such as stroke and Parkinson’s disease affect muscle activity and gait function during postural control [6], [33]. Therefore, D-COP_ML_ and V-COP_ML_ of the post-test might improve because participants of WBED groups could perform effective balance exercises that maintained their posture against unexpected perturbation by WBED. Moreover, the intervention test of our study and WBED perturbed participants laterally. Reactive postural control in response to unexpected lateral disturbances is mainly performed using the trunk and hip strategy, and these joint moments are responsible for the majority of postural control in response to lateral disturbances (approximately 85%) [29]. We assumed that perturbation-based exercise using WBED is effective in improving trunk and hip strategies by increasing muscle activity or response during reactive postural control.

Interestingly, although unexpected perturbation by WBED in the intervention session was in the mediolateral direction, COP_AP_ parameters in the WBED group significantly improved. In COP_AP_ parameters of the WBED group, D-COP_AP_ and RMS_AP_ of the post-test were significantly lower than those of the pre-test. The D-COP_AP_ owing to the unexpected perturbation also appeared from 0–0.4 s. A previous study reported that the older adults’ COP_AP_ parameters, such as RMS and mean velocity, during one-leg stance also increased with those of the COP_ML_ parameters [37]. Other previous studies had reported that older adults had increased COP displacement along with higher hip and trunk muscle activity during static and reactive postural control [8], [17]. Moreover, lateral perturbation caused higher EMG activity not only in the hip abductor and adductor muscles but also in the ankle dorsiflexor and plantarflexor muscles [38]. The results of COP_AP_ parameters in this study might be influenced by the activity of these muscles in the anteroposterior direction that is activated during unexpected lateral perturbation.

This study has several limitations. First, the number of participants of this study was only males and not large. A larger sample size may have provided more considerable results. Second, this study did not include older adults and individuals with impaired postural control. Future studies should include older adults and individuals with impaired postural control, with safety considerations. Furthermore, it is necessary to measure EMG and motion, as well as include questionnaire on balance exercise using wearable devices. Questionnaires may improve the usability of this device. Verification of the effectiveness of EMG and body motion might yield important insights in improving reactive postural control. Despite these limitations, perturbation-based balance exercise using WBED can improve reactive postural control function.

## V. Conclusion

This study aimed to investigate the effects of perturbation-based balance exercises performed using WBED on reactive postural control. The results showed that balance exercises using WBED decreased COP displacement during unexpected perturbation. Moreover, perturbation-based balance exercise using WBED improved reactive postural control function immediately. For fall prevention and the extension of a healthy livelihood, it is important to be able to perform perturbation-based balance exercises anywhere without instruments or medical expert guidance. Balance exercise using this device may help to reduce falls in older adults.
